# ORPHAcodes use for the coding of rare diseases: comparison of the accuracy and cross country comparability

**DOI:** 10.1186/s13023-023-02864-6

**Published:** 2023-09-04

**Authors:** Monica Mazzucato, Laura Visonà Dalla Pozza, Paola Facchin, Cèline Angin, Francis Agius, Clara Cavero-Carbonell, Virginia Corrochano, Katerina Hanusova, Kurt Kirch, Deborah Lambert, Caterina Lucano, Sylvie Maiella, Monica Panzaru, Cristina Rusu, Stefanie Weber, Oscar Zurriaga, Miroslav Zvolsky, Ana Rath

**Affiliations:** 1 RD Coordinating Centre, Veneto Region, Padua University Hospital, Padua, Italy; 2 French National Rare Disease Registry (BNDMR), Greater Paris University Hospitals (AP-HP), Paris, France; 3 Malta Mater Dei Hospital, Msida, Malta; 4 Rare Diseases Research Unit, Foundation for the Promotion of Health and Biomedical Research in the Valencian Region, Valencia, Spain; 5 CIBERER, Valencia, Spain; 6 Institute of Health Information and Statistics of the Czech Republic, Prague, Czech Republic; 7 BfArM, Bonn, Germany; 8 The Rotunda Hospital, Dublin, Ireland; 9 Inserm US14 – Orphanet, Paris, France; 10 Grigore T Popa-University of Medicine and Pharmacy, Iasi, Romania

**Keywords:** Rare diseases, Coding, Diagnoses, Orphanet, ORPHAcodes, ICD-10, Epidemiology, Public health

## Abstract

**Background:**

Estimates of rare disease (RD) population impact in terms of number of affected patients and accurate disease definition is hampered by their under-representation in current coding systems. This study tested the use of a specific RD codification system (ORPHAcodes) in five European countries/regions (Czech Republic, Malta, Romania, Spain, Veneto region-Italy) across different data sources over the period January 2019-September 2021.

**Results:**

Overall, 3133 ORPHAcodes were used to describe RD diagnoses, mainly corresponding to the disease/subtype of disease aggregation level of the Orphanet classification (82.2%). More than half of the ORPHAcodes (53.6%) described diseases having a very low prevalence (< 1 case per million), and most commonly captured rare developmental defects during embryogenesis (31.3%) and rare neurological diseases (17.6%). ORPHAcodes described disease entities more precisely than corresponding ICD-10 codes in 83.4% of cases.

**Conclusions:**

ORPHAcodes were found to be a versatile resource for the coding of RD, able to assure easiness of use and inter-country comparability across population and hospital databases. Future research on the impact of ORPHAcoding as to the impact of numbers of RD patients with improved coding in health information systems is needed to inform on the real magnitude of this public health issue.

## Introduction

Rare diseases (RD), in Europe defined as those with a prevalence of less than one per 2000, have progressively emerged as a global public health priority [1]. Their relevance relies on the fact that, although the number of patients diagnosed with a specific RD can be very low, the global population of persons living with a RD and in need of highly specialized health-care is far from negligible [2]. Despite the increasing recognition of rare diseases worldwide, there is a paucity of information regarding the magnitude of this relatively new medical concept and its impact at community level. The heterogeneity of the coding systems used in different countries and their general limited capacity of identifying RD patients in health information systems affect the availability of reliable data [3]. Several initiatives have been promoted at European and international level to tackle this issue. The adequate definition, codification and inventorying of RD were cited as priority areas of intervention in the Council Recommendation on an action in the field of rare diseases in 2009 [4]. In 2014, the Commission Expert Group on Rare Diseases adopted a “Recommendation on Ways to Improve Codification for Rare Diseases in Health Information Systems” [5]. In parallel with the process to incorporate codes for rare diseases in classification and coding systems as the International Classification of Diseases (ICD) and the Systematized Nomenclature of Medicine Clinical Terms (SNOMED-CT), the use of a specific RD coding resource was identified as a possible effective strategy to increase RD traceability in health information systems. To achieve this purpose, Orphanet has developed and continuously updates the Orphanet nomenclature of rare diseases, a multilingual standardized specific terminology dedicated to these conditions [6]. In order to be present in the Orphanet nomenclature, a disease, besides having a prevalence under the European rarity threshold (≤ 5 per 10,000), must be described in at least two independent individuals in the international scientific literature, confirming that it is not an incidental association of clinical signs [7]. In this nomenclature each clinical entity is assigned a unique and time-stable code, the ORPHAcode, around which the rest of the data present in the database is structured.

To incorporate a nosological level of representation of rare diseases, the Orphanet nomenclature has evolved into a hierarchical classification system (i.e. groups of disorders, disorders, and subtypes of a disorder) and per medical specialties. Given the multisystemic nature of many RD, each clinical entry can belong to one or more classifications and to one or more sections of a single classification (multiple parentage). In order to enable the sorting out of all clinical entities by medical specialty and avoid multiple counting of multi-classified entities in statistical analysis, each disease entity is assigned one classification group (called preferential parent) according to a defined procedure [8]. In order to assure interoperability across different information systems and data sources, the nomenclature is aligned with other international terminologies and reference databases (including ICD-10, ICD-11, SNOMED-CT, OMIM, UMLS, MeSH, MedDRA, and GARD to date) [9].

To tackle the RD under-representation issue in ICD-10, the World Health Organization (WHO) established in the context of the ICD revision process a Topic Advisory Group for rare diseases, managed by Orphanet [3]. After years of work, ICD-11 was adopted by the World Health Assembly in 2019 and came into effect on 1st January 2022. ICD-11 includes nearly 5500 rare diseases and their synonyms in the Foundation and aggregated under the same nonspecific morbidity and mortality statistics (MMS) code [10]. Nevertheless, the effects of the worldwide adoption of ICD-11 on morbidity and mortality statistics will not yet be visible for several years [11].

In the meantime, there is a growing interest in using a RD specific coding resource to improve patients’ visibility and foster data sharing across different care and research initiatives, including European Reference Networks for RD [12].

Growing efforts have been devoted to the alignment of data elements across data collections which is at the basis of data sharing and plays a critical role both in care and research initiatives [13, 14]. At European level the Platform on Rare Disease Registration (EU RD Platform) aims to address the fragmentation of rare disease (RD) patient data through the establishment of integration and interoperability standards. A set of 16 Common Data Elements (CDEs) for all RD registries has been identified, and highly recommends the use of ORPHAcodes to record RD diagnoses [15]. Furthermore, the implementation of ORPHAcodes in information systems has been recommended by the RARE 2030 foresight study and the European common semantic strategy, recognized as best practice by the Europe’s Steering Group on Promotion and Prevention of non-communicable diseases and a required data element for the European Patient Summary [16–18]. Currently, the Orphanet nomenclature of RDs contains over 6200 unique disorders, excluding groups of disorders and subtypes. Around 72% of them have a genetic basis and 84.5% are described by a prevalence of less than one case per million [2]. Few studies have been carried out using ORPHAcodes to provide insights into rare diseases epidemiology and to estimate their burden on the healthcare services [19–21]. Of note, these studies have been undertaken before a set of specific guidelines and rules for rare diseases coding was developed to ensure a homogenous approach and ensure intercountry data comparability. To further develop the Recommendations issued on RD coding, the Joint Action on Rare Diseases, RD-ACTION (2015–2018) [22], produced a tool set to assist European countries in implementing ORPHAcodes, including standard procedures and practical guidance for integration, use and routine maintenance of the Orphanet nomenclature in health information systems.

Specific guidelines for the use of ORPHAcodes for the coding of rare conditions have been developed [23]. The RD-CODE project (2019–2021), from which the current study arose, aimed to move forward by implementing ORPHAcodes into routine coding systems in four European countries (Malta, Romania, Czech Republic and Spain) to achieve a more accurate epidemiological surveillance of rare diseases. A further objective of the project was to collect feedback regarding the use of ORPHAcodes for coding purposes in different implementation settings, new and already operating ones, focusing both on hindering and facilitating factors. Based on these real-world implementation experiences, the developed coding resources and guidance documents for implementation were further refined, updated and made available to a wide range of potential users [24].

The aims of the present study are:

1. to test the easiness of use of the ORPHAcodes as a rare disease-specific coding resource across countries and regions participating in the RD-CODE project, evaluating their adaptability to different implementation settings;
2. to investigate the level of adherence to the RD coding guidelines developed so far;
3. to analyze the ORPHAcodes ability to describe rare diseases, in terms of corresponding aggregation level, prevalence class and ICD-10 alignment.
4. to estimate the added value of ORPHAcodes versus ICD-10 use to capture RD cases.

## Materials and methods

Data collection occurred in the frame of the RD-CODE project (www.rd-code.eu) during the period 1st January 2019–30th September 2021. Study populations were countries participating in ORPHAcodes implementation (Czech Republic, Malta, Romania and Spain). Spain participated as a consortium including six regional RD registries from the Basque Country, Castile and Leon, Navarre, Catalonia, Murcia and Valencian Region, covering around 40% of the Spanish population, the Rare Diseases Research Unit FISABIO-UVEG and the CIBERER. In addition, ORPHAcodes used in the same period by the RD population-based Registry of the Veneto region (Italy) were included in the present analysis [25]. Data ascertainment sources for each country are described in Table 1.

**Table 1:** Description of the study settings per country/region and number of ORPHAcodes collected during the study period

Country/region	N ORPHAcodes^b^	Data sources
Czech Republic	265	Congenital malformation registry/RD expert Centres
Malta	707	Congenital malformation registry/cancer registry/treatment abroad data
Romania	113	Genetic departments/RD expert Centres
Spain^a^	2378	RD regional population-based registries/RD expert Centres
Veneto region-Italy	1089	RD regional population-based registry/RD expert Centres

^a^Participating Consortium (RD regional registries of Basque Country, Castile and Leon, Navarre, Catalonia, Murcia and Valencia Region, the Rare Diseases Joint Research Unit FISABIO-UVEG and the CIBERER)

^b^Only ORPHAcodes corresponding to active RD entities in the Orphanet nomenclature (version July 2022) have been considered

As the focus of the study was the use of a RD specific coding resource, we did not consider the number and characteristics of registered patients per ORPHAcode. Thus, ORPHAcodes used in each country/region have been counted only once in global analyses.

We included in the analyses only ORPHAcodes assigned to patients with a confirmed RD diagnosis, as no specific ORPHAcode (ORPHA: 616874 “Rare disorder without a determined diagnosis after full investigation”) was available during the study period to allow the recognition of undiagnosed rare disease patients [26].

The nomenclature pack, annually released by Orphanet, includes a set of files produced to practically support the implementation of the Orphanet nomenclature in Health Information Systems [27]. For the present study we have referred to the 2022 version of the Orphanet nomenclature pack and in particular to the document “Description of the Orphanet nomenclature pack files for coding” to define the following concepts and the related analyses: ORPHAcodes, ORPHAcode aggregation, disease entity status (active/inactive), classification level (group of disorders, disorder or subtype of disorder), ICD-10 mapping relations [28].

We considered only ORPHAcodes corresponding to active rare diseases entities. We have excluded ORPHAcodes associated with inactive disease entities, described as those no longer present in the Orphanet nomenclature because they have become obsolete, deprecated or have been inactivated, as they cannot be considered rare according to the RD prevalence criterion in use in Europe (prevalence of no more than 5 per 10,000). For inactive entities, we have considered the ORPHAcode of replacement as assigned in the Orphanet nomenclature, when appropriate, and its corresponding aggregation level.

ORPHAcodes use according to the following aggregation levels was explored: group of disorders, disorder, subtype of disorder. When necessary for analysis, data with the classification level «subtype of disorder» was referred to the corresponding disorder level.

For the analyses of the ORPHAcodes per classification group, we considered the contents of the linearization file provided by Orphanet [8], in which a preferential medical specialty is attributed to every clinical RD entity. For the analysis of the ORPHAcodes per corresponding disease prevalence class, we considered the disease aggregation level and referred to prevalence values associated to each disease entity, as included in Orphadata applying the same methodology described in a previously published article [2].

ORPHAcodes associated to disorders whose point prevalence could not be calculated, such as those described by ‘prevalence at birth’, ‘lifetime prevalence’, or ‘annual incidence’ were excluded in the prevalence class analysis (n = 936). As in the countries/regions contributing to the study different versions of ICD classifications are in use for morbidity and mortality statistics, for homogeneity reasons we have considered cross-referenced ICD-10 codes derived from the Orphanet nomenclature pack (version 2022), given that ICD-10 is predominantly used for disease coding worldwide. Orphanet attributes ICD-10 codes to each ORPHAcode corresponding to a RD entity according to preestablished rules described in a procedural document, which is publicly available [28].

One of the aims of the study was to test the use of the ORPHAcodes as a coding resource intended to facilitate the identification of rare diseases, allowing a more accurate epidemiological surveillance and quantification of their burden. Thus, we aimed to estimate to which extent ORPHAcodes were able to better univocally describe RD compared to ICD codes. For this study purpose, we based our analyses on the alignment activity between ORPHAcodes and ICD-10 codes carried out by Orphanet, and made available in the Nomenclature pack [8]. The document defines how rare diseases included in the Orphanet nomenclature are aligned to, or attributed, a code in the World Health Organization’s International Classification of Diseases, 10th edition (ICD-10), as to whether the ORPHAcode is an exact match, is more precise, less precise or not clearly aligned to the ICD-10 code.

These relationships are described according to the following mutually exclusive categories:

- “Exact” when the ORPHAcode and the corresponding ICD-10 code describe the same disease entity;
- “Broader to narrower term (BTNT)” when the ORPHAcode has a broader range of application than the associated ICD-10 code;
- “Narrower to broader term (NTBT)” when the ORPHAcode has a narrower range than the ICD-10 code used to represent it;
- “not yet decided/unable to decide (ND)” when the alignment cannot be qualified by any of the preceding relationships.

We conducted descriptive analyses focused on the use of the ORPHAcodes per aggregation level of the Orphanet classification, disease prevalence class, preferential parent and Orphanet-ICD-10 alignment concept. Data analysis was centralized and conducted using SAS version 9.4 (SAS Institute, Cary, NC). No ethical approval was sought or required for this project, as it did not involve the collection of data regarding individuals. De-identified lists of ORPHAcodes with no patient identifiers were generated by participating centers and transferred to investigators for aggregate analysis.

## Results

During the study period 4552 ORPHAcodes were used to record RD patients across the 5 different settings and the ascertainment sources considered. The number of the ORPHAcodes used reduces to 3133, after removing duplicates and considering only active entities. The contribution of the different participating countries/regions to the data collection per ORPHAcodes aggregation level is presented in Table 2. The vast majority of the ORPHAcodes used during the study period correspond to the disorder level of aggregation of the nomenclature (n = 2200; 70.2%), whilst 557 (17.8%) and 376 (12%) referred to groups of disorders and to subtypes of disorders respectively. This distribution was consistent across all settings. When ORPHAcodes assigned to subtypes of disorders were referred to the corresponding disorder level, overall 82.2% of the ORPHAcodes used were able to describe entities at the disorder level.

**Table 2:** Distribution of ORPHAcodes collected during the study period per country/region and per aggregation level of the Orphanet classification

Country/region	Group of disorders		Disorder		Subtype of disorders	
	N	%	N	%	N	%
Czech Republic	31	11.7	192	72.5	42	15.8
Malta	113	16.0	536	75.8	58	8.2
Romania	15	13.3	97	85.8	1	0.9
Spain	378	15.9	1719	72.3	281	11.8
Veneto region-IT	223	20.5	740	68.0	126	11.5
All countries/regions^a^	557	17.8	2200	70.2	376	12.0

^a^ORPHAcodes used in more than one country/region have been considered only once in the global analysis

**Fig. 1:**
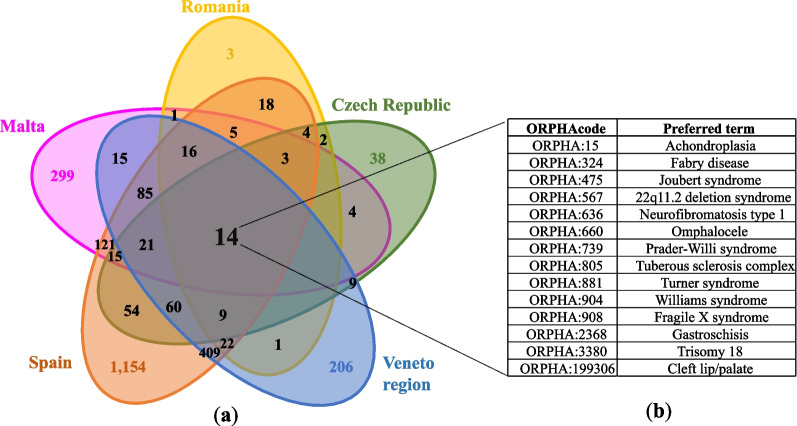
Distribution of ORPHAcodes used and their overlap between country/regions of the study (**a**); ORPHAcodes used in all the settings described by the preferred term used in the Orphanet nomenclature version 2022 (n = 14) (**b**)

The overlap of ORPHAcodes used in the different contributing countries/regions and corresponding to a disorder aggregation level is represented in Fig. 1. The settings in which the greatest overlap of ORPHAcodes used to record rare patients occurred are Spain and the Veneto region, Italy (n = 409) representing 17.1% and 37.5% of all the ORPHAcodes used in these two study areas, respectively. A core of 14 ORPHAcodes was used in all the five study settings. They correspond mainly to chromosomal anomalies, multiple congenital anomalies and syndromes, all of which have as preferential parent the Orphanet classification of Rare developmental anomalies during embryogenesis (Fig. 1b).

**Fig. 2:**
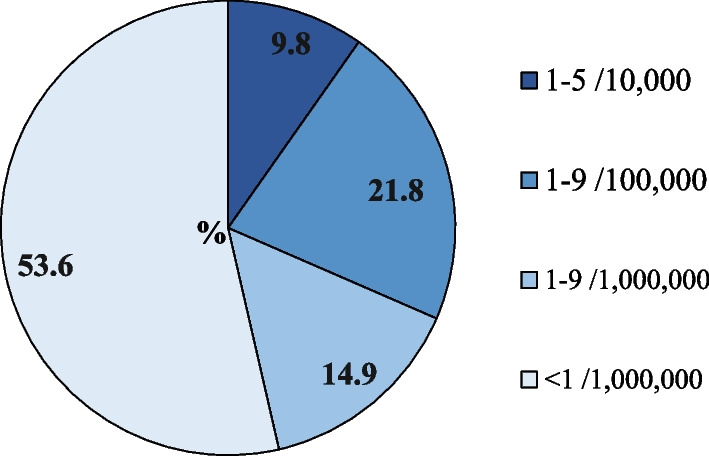
Distribution of ORPHAcodes collected during the study period per disease prevalence class. The value of the disease prevalence class has been attributed to ORPHAcodes for which a reported prevalence was available in the Orphanet nomenclature version 2022 (n = 1640) and according to the method described in [2]

For ORPHAcodes for which a reported prevalence is available (n = 1640), more than half (n = 879; 53.6%) have a reported prevalence of less than 1 case per million (Fig. 2).

An additional 14.9% are ORPHAcodes corresponding to rare diseases described by a prevalence of 1–9 cases per million. A small proportion of ORPHAcodes (9.7%) were diseases falling in the prevalence class closest to the European rarity threshold (i.e. 1–5 per 10,000). Distribution of the ORPHAcodes used per country/region and disease prevalence class is shown in Table 3, with Spain and Veneto region, Italy, having the great proportion of ORPHAcodes used falling in the lowest prevalence classes, respectively 51.8% and 37.3%. Of note, diseases excluded in this analysis because their prevalence is unknown (n = 936) most likely represent ultra-rare diseases with prevalence of less than per million for which epidemiological data are not available.

**Table 3:** Distribution of ORPHAcodes collected during the study period per country/region and per disease prevalence class^a^

Disease prevalence class	Czech Republic		Malta		Romania		Spain		Veneto region-IT		All countries/regions	
	N	%	N	%	N	%	N	%	N	%	N	%
1–9/10,000	29	16.1	77	22.6	23	27.1	135	10.1	66	10.0	160	9.7
1–9/100,000	69	38.3	117	34.3	31	36.5	299	22.3	203	30.9	357	21.8
1–9/1,000,000	39	21.7	51	15.0	15	17.6	212	15.8	143	21.8	244	14.9
< 1/1,000,000	43	23.9	96	28.1	16	18.8	695	51.8	245	37.3	879	53.6
Total	180	100	341	100	85	100	1341	100	657	100	1640	100

^a^The value of the disease prevalence class has been attributed to ORPHAcodes for which a reported prevalence was available in the Orphanet nomenclature (version July 2022) and according to the method described in [2]

**Fig. 3:**
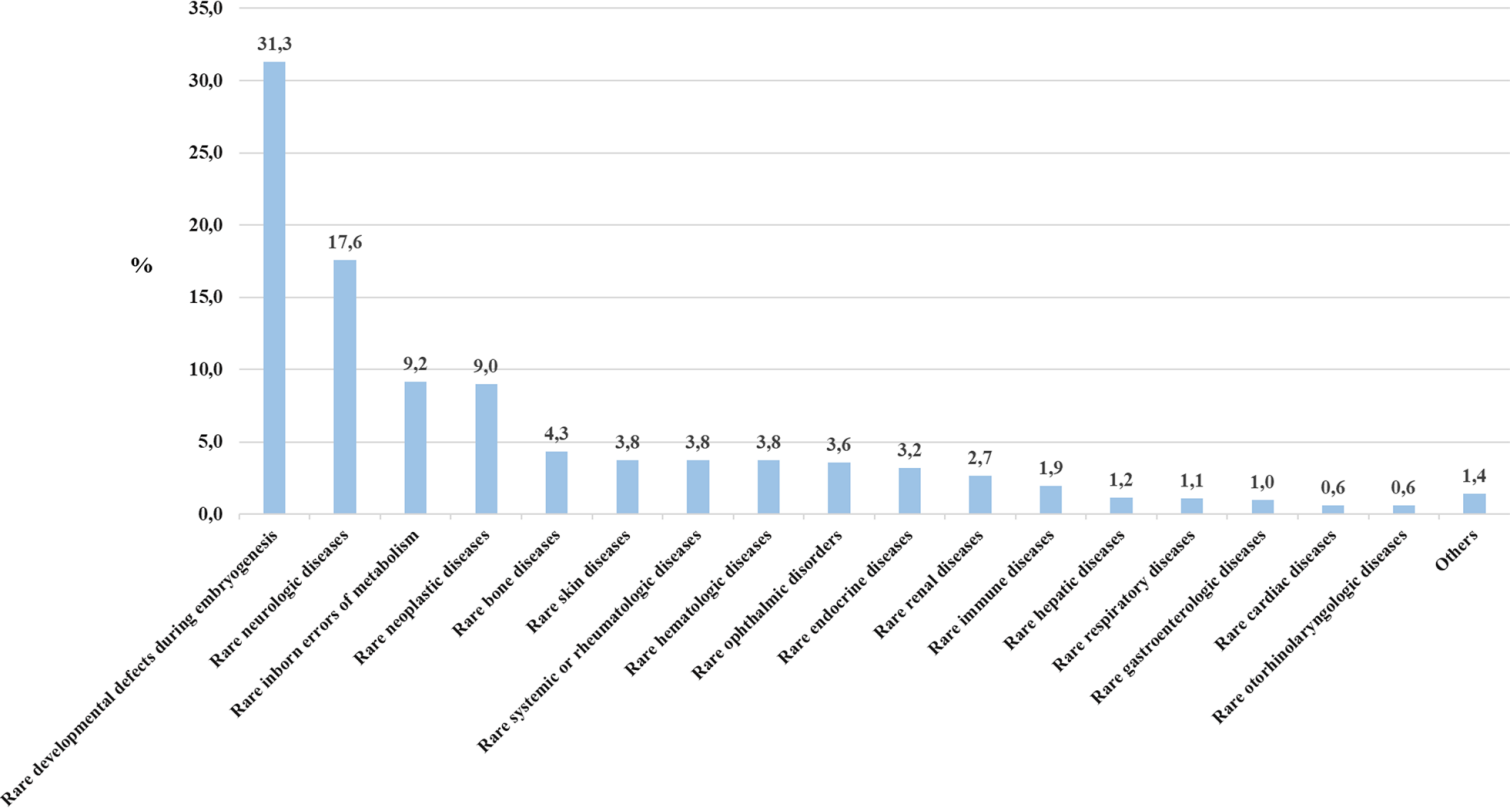
Distribution (%) of ORPHAcodes collected during the period (1st January 2019–30th September 2021) in all countries/regions by preferential parent of the Orphanet classification (version 2022) in decreasing order of frequency (n = 2576)

ORPHAcodes reported encompassed the whole spectrum of the Orphanet classifications, most commonly the rare developmental defects during embryogenesis (31.3%) and the rare neurological diseases (17.6%). In addition, ORPHAcodes captured inborn errors of metabolism (9.2%), neoplastic conditions (9.0%) and, to a lesser extent, diseases belonging to almost all the other remaining Orphanet classifications (Fig. 3).

With regards to the comparison of ORPHAcodes to ICD-10 codes, the distribution of ORPHAcodes per country/region of use and alignment concept is presented in Table 4. In all the study settings, the great majority of ORPHAcodes used is described by the relationship “Narrower to broader term”, considering the corresponding aligned ICD-10 code. Overall, 83.4% of the ORPHAcodes used described their disease associated entity more precisely than the corresponding ICD-10 code.

**Table 4:** Distribution of ORPHAcodes collected during the study period per country/region and per ICD-10 alignment concept as assigned by Orphanet (version 2022)

Alignment concept	Czech Republic		Malta		Romania		Spain		Veneto region-IT		All countries/regions^a^	
	N	%	N	%	N	%	N	%	N	%	N	%
E (Exact mapping)	39	2.1	108	3.4	15	3.1	214	3.2	94	1.6	257	10.0
BTNT (ORPHAcode's Broader Term maps to a Narrower Term)	10	16.7	49	18.2	8	15.3	38	10.7	17	10.9	79	3.1
NTBT (ORPHAcode's Narrower Term maps to a Broader Term)	180	76.9	417	70.2	72	73.4	1684	84.2	741	85.5	2149	83.4
ND (not yet decided/unable to decide)—missing	5	4.3	20	8.2	3	8.2	64	1.9	14	2.0	91	3.5
Total	234	100	594	100	98	100	2000	100	866	100	2576	100

^a^ORPHAcodes used in more than one country/region have been considered only once in the global analysis

**Fig. 4:**
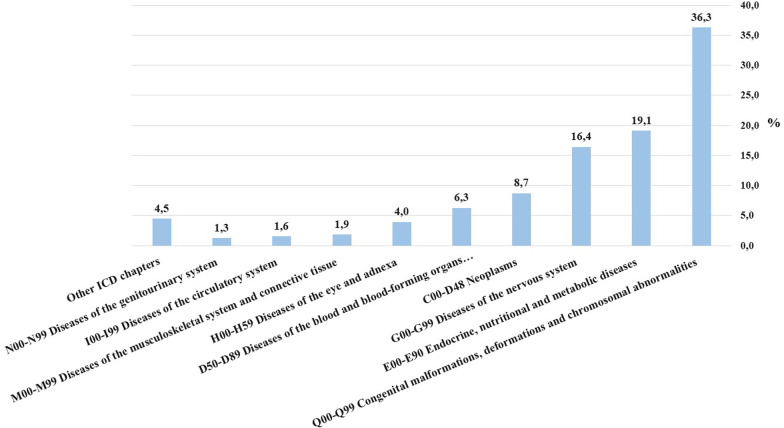
Distribution of ORPHAcodes having a Narrower to Broader Term (NTBT) ICD-10 mapping in the Orphanet nomenclature pack (version 2022) by ICD corresponding chapter in increasing order of frequency (n = 2149)

The distribution of ICD-10 codes related to the ORPHAcodes alignment correspondence “Narrower to broader term” (NTBT) is represented in Fig. 4. Most of these codes fall into the following ICD-10 chapters: “Q” (36.3%), “Congenital malformations, deformations and chromosomal abnormalities”; “E” (19.1%), “Endocrine, nutritional and metabolic diseases”, and “G” (16.4%), “Diseases of the nervous system”. This again illustrates ORPHAcodes’ ability to more accurately describe rare diseases entities compared to ICD is particularly evident in these three disease domains.

## Discussion

This is the first study describing ORPHAcodes use in five European countries, based on data coming from real-world implementation settings. Our findings confirmed the value of the multilingual nature of the Orphanet nomenclature and of ORPHAcodes as a common exploitable coding resource, beyond language and country specificities. The study highlighted that ORPHAcodes have been used mainly to record diagnoses referred to the disorder level of aggregation of the Orphanet nomenclature of RD. This is in line with the classification granularity level recommendations issued by the RD-ACTION [23]. Where ORPHAcodes corresponding to groups of diseases have been used, for instance for partial chromosome deletions, this probably reflects a different diagnostic capacity across participating regions. Another case in which classification as groups occurs is where national mandatory RD monitoring lists include some group entities as is the case of Italy [29]. The study demonstrated that ORPHAcodes comparability in terms of distribution per aggregation level and preferential classification increases when they are used in the context of population-based, rather than hospital-based data collections, as in Spain and the Veneto region, Italy.

Our study has shown that ORPHAcodes improve visibility of RD in health information systems Notably, ORPHAcodes use allows to improved capture of ultra-rare diseases, which are under-represented in ICD-10. More than the 85% of the ORPHAcodes used during the study period were able to better univocally describe individual rare diseases entities than the corresponding ICD-10 codes. Moreover, diseases falling into the three following ICD-10 chapters seemed to benefit the most from ORPHAcoding: “Q”—congenital malformations, deformations and chromosomal abnormalities; “E”—endocrine, nutritional and metabolic diseases and “G”—diseases of the nervous system. This finding of a higher agggregation of ORPHAcodes in these chapters confirms the results of a recent Spanish study assigning ICD-10-ES codes equivalencies to a consistent proportion of ORPHAcodes (n = 5664) [30].

Despite diversities of the implementing countries/regions in terms of health-care organization, coding system used to record morbidity data, settings in which ORPHAcodes have been used, personnel involved, IT systems used for data collection and languages used to record RD diagnoses, ORPHAcodes were considered by users to be a versatile coding resource, which can be effectively introduced in different settings preserving consistency. The creation of a community of practice was suggested to move forward in the process of RD coding to increase patients’ visibility across diverse health-care settings [31].

## Limits of the study

The present study presents some limitations which deserve to be mentioned. First, the study period was limited in time, considering that the coding resource evaluated has been developed to capture rare diseases, for which a long observation period is usually needed to identify cases, especially ultra-rare ones. This limit is partially mitigated by the wide geographic capture of the study, involving different implementation settings in five European countries/regions. Despite this, it is clear that the nature of the ORPHAcodes analyzed is highly dependent on the data source considered, both in terms of which are the monitored conditions and of which is the setting where the data collection took place. Population-based registries ongoing in Spain and the Veneto region (Italy) contributed the most in terms of variety of collected ORPHAcodes and representation of the whole spectrum of corresponding rare disease entities. Nevertheless, the snapshots offered by other implementation settings, as genetic departments and RD expert Centres in the Czech Republic and Romania, and by other population-based registries, although with smaller catchment areas, such as the cancer registry in Malta, have contributed as well to the study purposes, being instrumental to demonstrate the versatility of use of the coding resource under study.

A further limit of the present study is that we analyzed only ORPHAcodes able to describe confirmed rare diseases cases, without considering patients with suspected rare diseases or undiagnosed patients. To tackle this issue the RD-CODE project has developed an operational definition of undiagnosed patient, namely a patient without a determined diagnosis after full investigation. A new ORPHAcode has been created (ORPHA: 616874) to encourage the recognition of undiagnosed rare disease patients as a distinct population with specific unmet health and social care needs. As this code has been introduced only recently in the Orphanet nomenclature and was not in use during the study period, we focused on confirmed RD diagnoses. Is it worth making a mention that both upskilling of professionals in countries and data recording was affected by COVID restrictions on gatherings and in some cases the reassignment of staff to COVID population health services.

Given the limited period of the study and the heterogeneity of the data sources considered, either population-based and centre-based ones, we were not able to quantify the impact of ORPHAcoding in terms of the number of patients with improved coding by our study methodology. However, we can hypothesize a considerably increased ability to identify RD patients, based on the findings available from population-based studies which identified these disease groups as the ones contributing the most to the RD population [19, 20, 32]. Further studies will be needed to exactly quantify the added value of the use of the ORPHAcodes in tracing rare disease patients in health information systems.

## Conclusions

Despite the above-mentioned limitations, this study sets the basis for a widespread use of ORPHAcodes to record patients’ diagnoses across different rare diseases data collections. The use of the ORPHAcodes in health records and patient registries according to the coding supporting tools developed by the RD-CODE project, in which the current study is framed, can ensure that RD data are collected correctly and uniformly across countries, despite the different terminologies and classifications systems in use. Recognizing RD as a public health priority, data are needed in guiding health planning and clinical service delivery and are furthermore instrumental for the monitoring of all the initiatives put in place. Health data annotated with ORPHAcodes can be used to collect more precise data on rare disease patients, representing the multifaceted nature of these complex conditions, presenting peculiar research and care needs. This is a necessary step to create a common space where information about rare diseases can be shared by policy-makers, clinicians, researchers, industry and patients to build on the achievements of the last decades and to maintain the focus on this unique public health challenge.

## Data Availability

The data that support the findings of this study are available from the corresponding authors [MM, AR], upon motivated request.
